# Case Report: Meningioma with unusual extracranial extension to the temple and orbit

**DOI:** 10.3389/fonc.2026.1698840

**Published:** 2026-01-30

**Authors:** Jing Bao, Xuxu Xu, Zhenjiang Pan, Shepeng Wei

**Affiliations:** Department of Neurosurgery, Shanghai Shidong Hospital of Yangpu District, Shanghai, China

**Keywords:** extracranial extension, meningioma, orbital involvement, stereotactic radiosurgery, subcutaneous temporal mass

## Abstract

Meningiomas are the most common primary intracranial tumors, yet extracranial extension occurs in only 1% to 2% of cases and is rarely the initial manifestation. We report a 66-year-old man who presented with a progressively enlarging right frontotemporal subcutaneous mass that had recently accelerated in growth and was initially considered a benign soft tissue lesion. Computed tomography revealed a subcutaneous mass with additional lesions of similar attenuation involving the intracranial and intraorbital compartments. Contrast-enhanced magnetic resonance imaging demonstrated homogeneously enhancing lesions in the subcutaneous temporal tissue, temporalis muscle, orbit, and the floor of the middle cranial fossa, raising concern for a common origin with multicompartment extension. The extracranial component was excised under general anesthesia; the mass was located deep to the temporalis muscle and was removed completely with partial preservation of the muscle. Histopathological examination confirmed a World Health Organization grade I meningothelial meningioma with a Ki-67 labeling index of 10%. Early postoperative CT obtained 1 week after surgery showed expected postoperative changes and confirmed that the intraorbital and middle cranial fossa lesions had not been treated. At telephone follow-up, the patient reported no neurologic or visual symptoms; he had traveled abroad and elected to defer further treatment and surveillance imaging, with plans to re-evaluate after returning in approximately 6 months. This case illustrates an unusual growth pattern of meningioma with simultaneous intracranial, subcutaneous, muscular, and orbital involvement presenting primarily as a temporal mass with minimal neurologic or ophthalmologic symptoms. Comprehensive neuroimaging was essential for diagnosis and mapping of disease extent, and management required balancing surgical accessibility, anticipated control with radiosurgery, and patient preference within a multidisciplinary framework.

## Introduction

Meningiomas are the most common primary intracranial tumors and account for a substantial proportion of central nervous system neoplasms. Although they typically present with neurologic signs related to mass effect or cranial nerve involvement, their clinical manifestations can be variable and occasionally misleading. We report an unusual presentation of a meningioma that initially manifested as a temporal subcutaneous mass without classic neurologic or ophthalmologic symptoms. This case underscores the importance of considering an intracranial-origin meningioma in the differential diagnosis of extracranial temporal masses, particularly when imaging suggests multicompartment involvement.

## Case presentation

**Figure 1:**
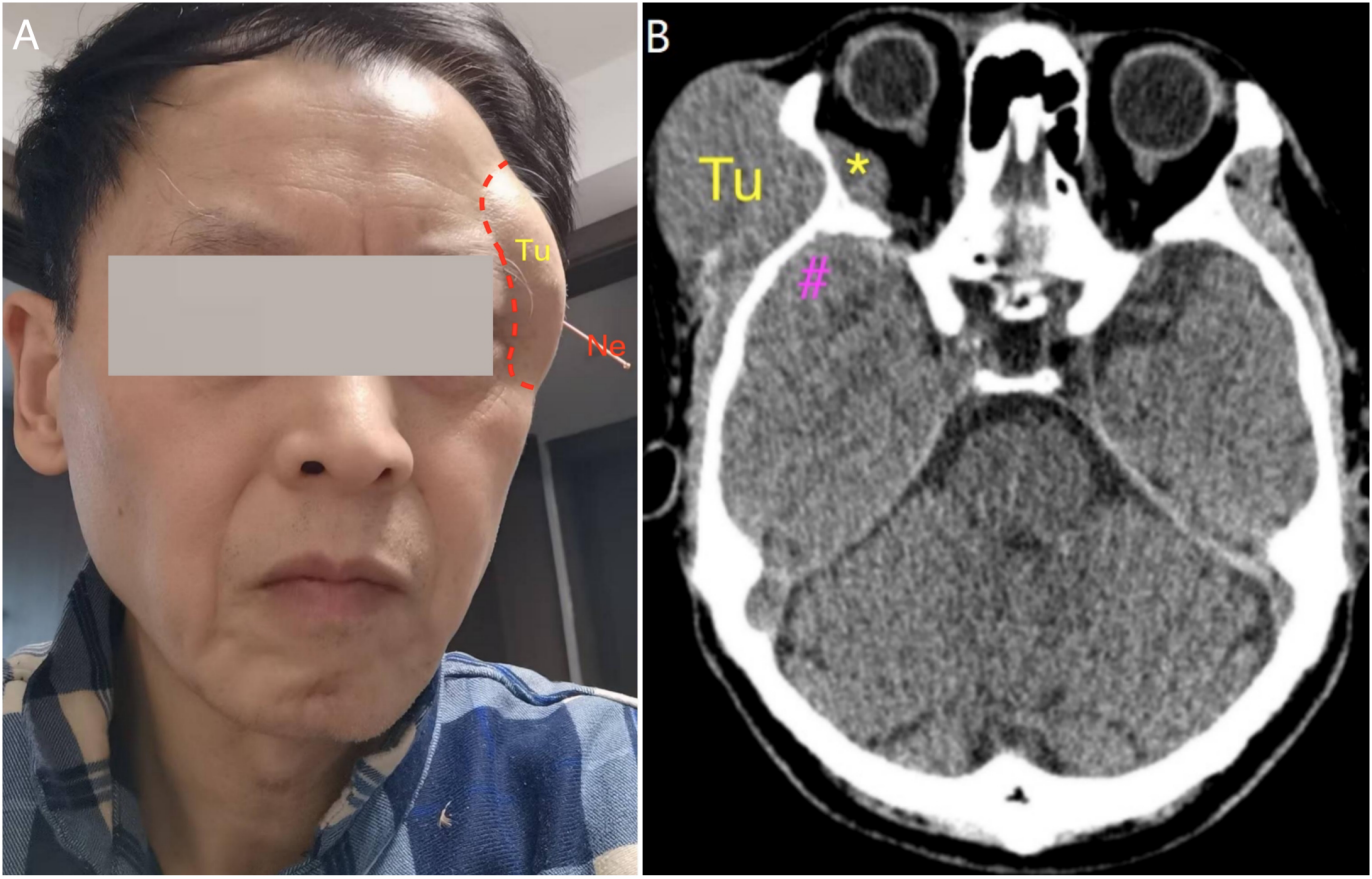
Clinical photograph and preoperative non-contrast CT. **(A)** Clinical photograph showing the right frontotemporal subcutaneous mass; the patient had previously undergone acupuncture. **(B)** Representative axial non-contrast head CT demonstrating lesions in the frontotemporal subcutaneous tissue (Tu), the orbit (*), and the floor of the middle cranial fossa adjacent to the temporal pole (#); retained acupuncture needles are visible (Ne). Tu, tumor; *, intraorbital lesion; #, middle cranial fossa lesion; Ne, retained acupuncture needles.

A 66-year-old man presented with a 2-year history of a right frontotemporal subcutaneous mass that had enlarged more rapidly over the preceding month. He first noticed a rice-grain-sized nodule in the right temple 2 years earlier; the lesion gradually enlarged to approximately quail egg size and continued to progress, measuring approximately 5.5 × 5.0 cm at presentation with a broad base. At 2 months before admission, he underwent acupuncture for 2 weeks and discontinued it because of no perceived benefit (**Figure 1**). Relevant past medical history, medication use, allergies, family history, and other psychosocial details were not fully documented in the available record; however, subsequent decision-making was strongly influenced by the patient’s preference to defer further treatment because of impending international travel.

On examination, the overlying skin was intact, and there was mild localized tenderness without erythema, ulceration, or substantial pain. The neurologic examination was unremarkable, with no focal deficits. Ophthalmologic assessment revealed normal visual acuity and visual fields, and no diplopia, ocular motility limitation, or other orbital symptoms were reported.

**Figure 2:**
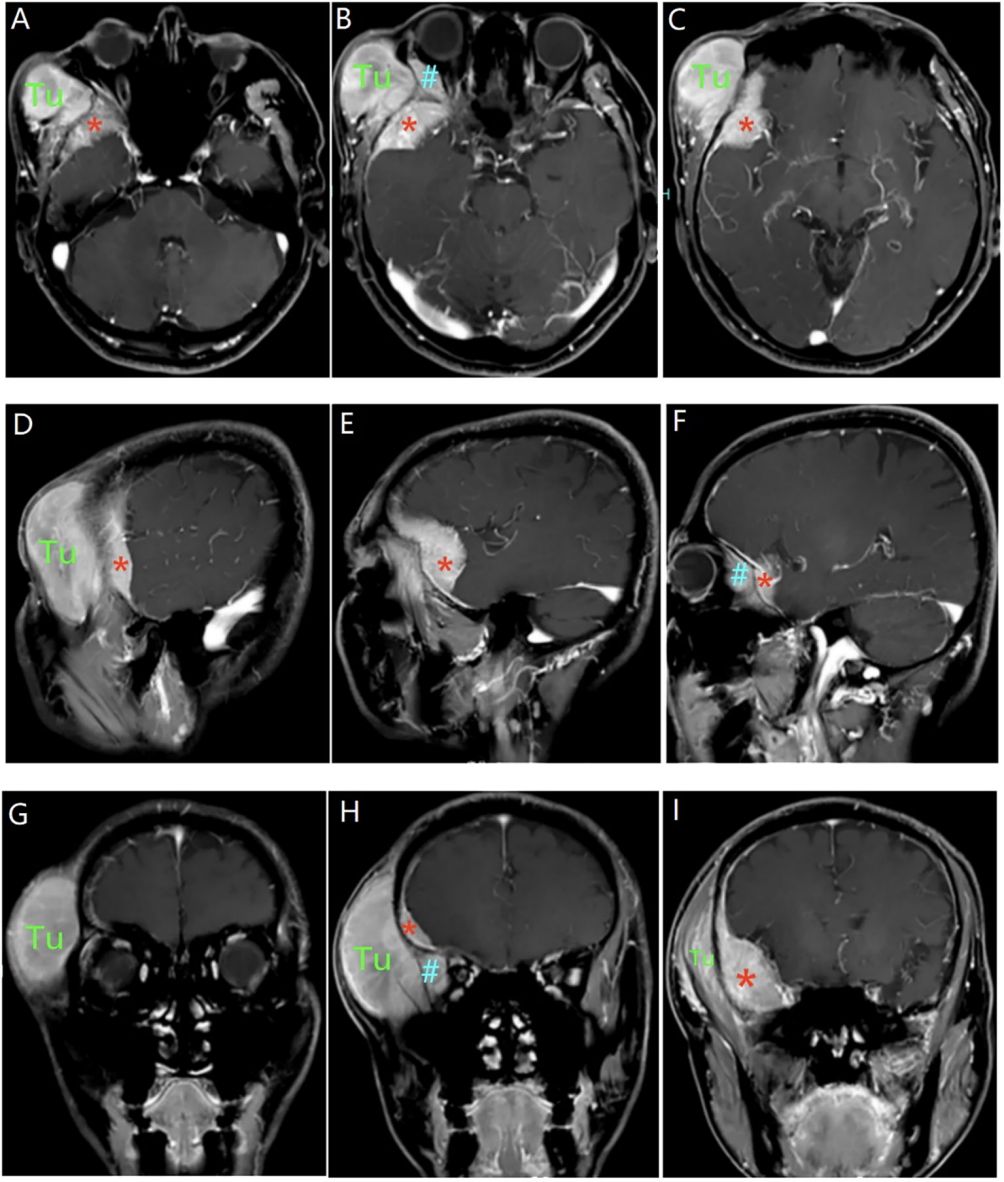
Preoperative post-contrast T1-weighted MRI in multiple planes. Representative post-gadolinium T1-weighted MRI shows homogeneously enhancing lesions involving the right frontotemporal subcutaneous tissue (Tu), orbit (*), and the floor of the middle cranial fossa (#) on axial **(A–C)**, sagittal **(D–F)**, and coronal **(G–I)** images. Tu, tumor; *, intraorbital lesion; #, middle cranial fossa lesion.

**Figure 3:**
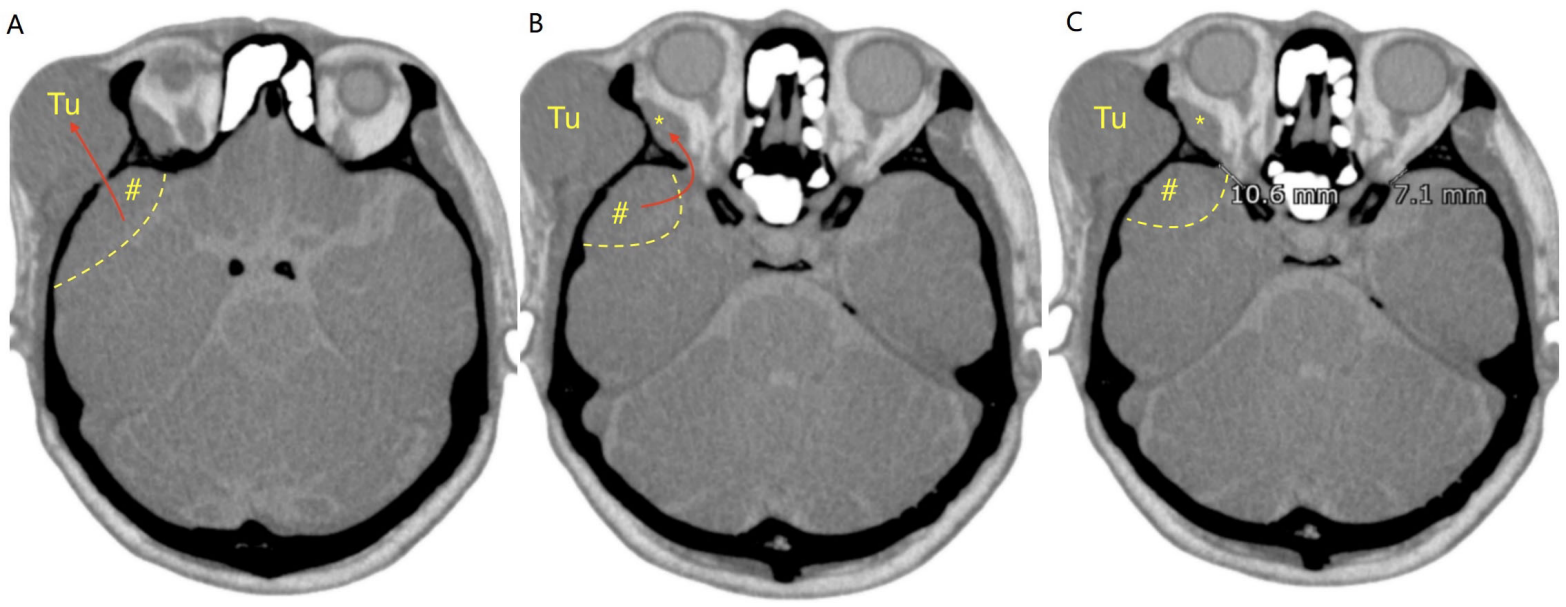
Preoperative bone-window CT demonstrating inferred routes of extension. **(A)** Axial bone-window CT showing a lesion centered at the floor of the middle cranial fossa (#); the long arrow indicates a suspected transosseous/foraminal route toward the extracranial temporal compartment. **(B)** Tumor extension into the orbit through an enlarged superior orbital fissure (arrow). **(C)** Right-to-left asymmetry with enlargement of the right superior orbital fissure. *, intraorbital lesion; #, middle cranial fossa lesion.

Head CT demonstrated a right frontotemporal subcutaneous mass and suggested additional lesions of similar attenuation in the intraorbital compartment and at the floor of the middle cranial fossa adjacent to the temporal pole (**Figure 1**). Contrast-enhanced MRI showed homogeneously enhancing lesions involving the frontotemporal subcutaneous tissue, temporalis muscle, orbit, and the middle cranial fossa floor (**Figure 2**), raising concern for a common origin with multicompartment extension. Preoperatively, the site of origin was uncertain. Bone-window CT later supported transosseous or foraminal extension from the middle cranial fossa floor toward the extracranial compartment and orbital involvement through an enlarged superior orbital fissure (**Figure 3**).

### Therapeutic intervention

**Figure 4:**
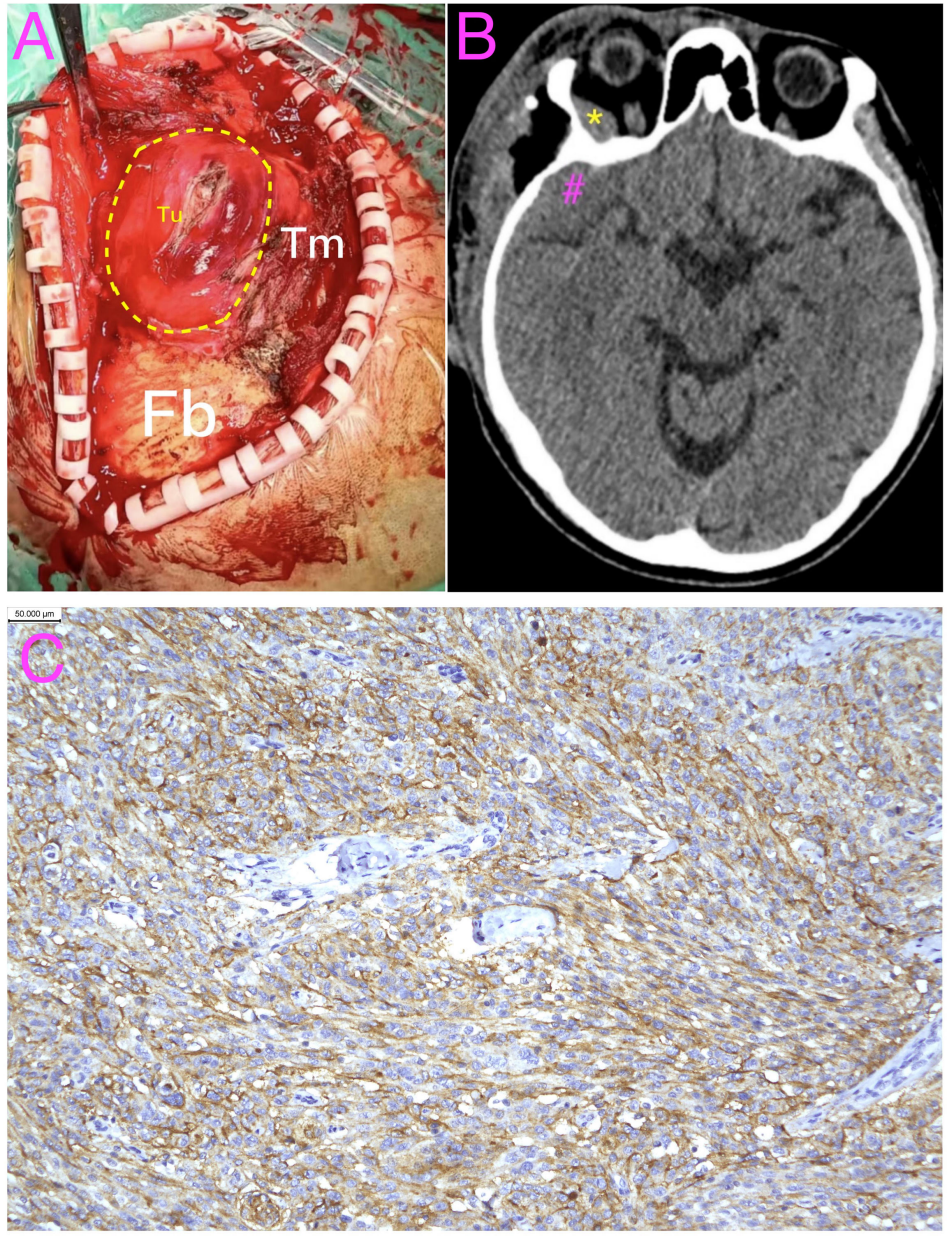
Intraoperative findings, postoperative CT, and histopathology. **(A)** Intraoperative view showing the main tumor mass (Tu) located deep into the temporalis muscle (Tm) and external to the cranial bone (Fb). **(B)** Representative axial non-contrast head CT obtained on postoperative day 7 showing expected postoperative changes and confirming that the intraorbital (*) and middle cranial fossa (#) lesions were not treated. **(C)** Ki-67 immunohistochemistry demonstrating a labeling index of approximately 10% (scale bar, 50 μm). Tu, tumor; Tm, temporalis muscle; Fb, frontal bone; *, intraorbital lesion; #, middle cranial fossa lesion.

The patient underwent excision of the extracranial component under general anesthesia. The mass was located deep to the temporalis muscle with a well-circumscribed plane and limited vascularity (**Figure 4**). The extracranial lesion was removed completely with partial preservation of the temporalis muscle. At the patient’s request, the intracranial and intraorbital lesions were not surgically addressed; the residual soft tissue within the temporal bone defect was coagulated. The estimated blood loss was minimal.

### Histopathology

Histopathologic examination confirmed an epithelial (meningothelial) meningioma (CNS WHO grade 1). Immunohistochemistry showed CKpan negativity and diffuse expression of EMA and vimentin, supporting a meningioma phenotype. The tumor demonstrated SSTR2 positivity and GFAP and CD34 negativity. Progesterone receptor (PR) expression was focal/variable (±). Additional markers showed H3K27me3 (±), E-cadherin positivity, D2–40 negativity, p16 positivity, and MTAP negativity (loss of expression). The Ki-67 labeling index was approximately 10%, indicating intermediate proliferative activity within the context of CNS WHO grade 1 histology.

### Follow-up and outcomes

The patient was advised to consider stereotactic radiosurgery for the residual disease and to undergo interval imaging surveillance. At subsequent telephone follow-up, the patient reported that he felt well without neurologic or visual symptoms; he had traveled abroad and elected to defer further treatment and follow-up imaging, with plans to re-evaluate after returning in approximately 6 months. No adverse or unanticipated events were reported during the available follow-up period.

### Patient’s perspective

The patient reported that the temporal mass was his primary concern and that he felt well after surgery. He expressed a preference to defer additional treatment and surveillance imaging while abroad and planned to reconsider management options after returning in approximately 6 months.

### Clinical timeline

2 years before admission: Noticed a small right temporal nodule; gradual enlargement over time.

2 months before admission: Acupuncture for 2 weeks; discontinued due to lack of improvement.

1 month before admission: Accelerated growth of the temporal mass.

Admission: CT and contrast-enhanced MRI demonstrated multicompartment lesions.

Surgery: Excision of the extracranial component; intracranial and intraorbital lesions were left untreated per patient preference.

Postoperative day 7: Head CT showed expected postoperative changes; the residual intraorbital and middle cranial fossa lesions remained untreated.

Follow-up: Telephone follow-up—patient asymptomatic; traveled abroad; deferred further treatment and surveillance imaging; planned re-evaluation after ~6 months.

## Discussion

Meningiomas are the most common primary intracranial tumors and account for approximately 30% of all brain tumors (1). Extracranial extension is uncommon, occurring in only 1% to 2% of cases (3, 4).

What this case adds: The present case expands the limited literature on meningiomas with extracranial extension by demonstrating a rare synchronous multicompartment pattern involving the temporal subcutaneous tissue, temporalis muscle, orbit, and the floor of the middle cranial fossa while presenting primarily as a temporal subcutaneous mass with minimal neurologic or ophthalmologic symptoms. This presentation differs from the more typical spheno-orbital meningioma phenotype, in which proptosis, visual impairment, and ocular paresis often predominate (7, 8), and underscores a potential diagnostic pitfall when an apparently benign temporal soft tissue mass is the chief complaint despite substantial intracranial and orbital disease. To better contextualize this rare synchronous multicompartment pattern and to address the key differential diagnosis of primary extracranial meningioma, we summarized selected relevant reports in the literature (**Table 1**).

**Table 1:** Selected literature context for meningioma with extracranial extension to the temporal region and/or orbit and differential context of primary extracranial meningioma.

Row/source	What it represents (why it is here)	Key overlap with our case	Typical management/outcome reported (high level)
Present case (this report)	Synchronous multicompartment involvement presenting as a temporal subcutaneous mass with minimal symptoms	Subcutaneous temporal + temporalis muscle + orbit + skull-base/middle cranial fossa floor involvement; mild/absent neuro-ophthalmic symptoms; intracranial origin supported by bone-window CT features (inferential)	Excision of extracranial component; intracranial/orbital lesions left *in situ* per patient preference; SRS recommended but not performed; short-term POD7 CT; telephone follow-up asymptomatic
Selected reports of extensive spheno-orbital/skull-base meningioma with marked extracranial/orbital extension (3, 5, 9, 11)	“Closest neighbors” in the literature that support feasibility/precedent of extensive extracranial and/or orbital extension	Orbital involvement and/or extensive extracranial growth pattern (including skull-base routes and extracranial extension)	Management varies but commonly includes surgery for accessible components and consideration of adjuvant radiotherapy/radiosurgery depending on residual disease and setting
Primary extracranial meningioma (differential context) (4)	Addresses reviewer’s concern: intracranial origin vs. primary extracranial meningioma	Extracranial location can mimic benign soft tissue lesions; diagnosis often relies on pathology and careful exclusion of intracranial primary	Generally treated with excision; follow-up recommended; serves as key alternative diagnosis when extracranial mass is the presenting complaint

Extracranial extension of meningioma has been proposed to occur through natural foramina, direct transosseous invasion, or along vascular channels (5, 6). In this patient, the overall imaging pattern was most consistent with an intracranial dural-based origin with secondary extracranial and orbital extension. Although the precise site of origin cannot be proven radiologically, several imaging features supported an intracranial origin (inferential): (1) a lesion centered at the floor of the middle cranial fossa on bone-window CT, (2) an apparent transosseous/foraminal route extending from the middle cranial fossa toward the extracranial temporal compartment, and (3) orbital involvement through an enlarged superior orbital fissure with right-to-left asymmetry. In addition, the homogeneous enhancement pattern across compartments on contrast-enhanced MRI favored a single unifying meningiomatous process rather than metastatic disease (2).

The growth pattern in this case is notable. Spheno-orbital meningiomas are commonly characterized by prominent orbital symptoms related to intraorbital involvement (7, 8). In contrast, our patient’s dominant symptom was a temporal subcutaneous mass with minimal orbital manifestations, a presentation that has only rarely been emphasized in the literature (9). The coexistence of subcutaneous, muscular, and orbital components also posed diagnostic challenges and reinforced the importance of comprehensive imaging to delineate the full extent of the disease and to guide management (2, 6). While CT is valuable for assessing osseous remodeling and defining potential transosseous routes, contrast-enhanced MRI is essential for mapping soft tissue extent and identifying synchronous enhancing lesions consistent with a shared origin (2).

The differential diagnosis for a temporal mass with multicompartment involvement includes primary extracranial meningioma, metastatic disease, sarcoma, and other soft tissue neoplasms (4, 5). In this case, the synchronous intracranial and intraorbital lesions, together with homogeneous enhancement across compartments and a meningioma-consistent immunophenotype, supported a single meningiomatous process rather than multifocal metastasis or a primary extracranial malignancy (2).

Management of meningiomas with extracranial extension requires balancing surgical accessibility, anticipated disease control with adjuvant radiotherapy/radiosurgery, and patient preference. Complete surgical excision is generally favored for accessible symptomatic components, whereas skull-base, intracranial, or orbital disease may require individualized decision-making to minimize morbidity while achieving durable local control (7, 8, 10). In the present case, the extracranial component was removed completely, while the intracranial and intraorbital lesions were left *in situ* at the patient’s request. Stereotactic radiosurgery was recommended as a noninvasive option for the residual intracranial and intraorbital lesions given the multicompartment distribution and the patient’s preference to avoid additional surgery, consistent with published experience (8, 11). However, it was not performed because the patient traveled abroad and elected to defer further treatment and surveillance imaging, with plans to re-evaluate after returning in approximately 6 months.

In summary, this case highlights that meningioma should remain in the differential diagnosis of temporal soft tissue masses, particularly when imaging suggests multicompartment continuity involving the skull base and orbit. It also illustrates the value of combining bone-window CT with contrast-enhanced MRI to infer potential routes of extracranial extension and to map the disease extent, and it emphasizes the need for individualized management within a multidisciplinary framework for complex multicompartment presentations.

## Conclusion

We describe a rare case of intracranial meningioma with extensive extracranial extension involving the subcutaneous temporal region, temporalis muscle, and orbit. This case emphasizes the importance of including meningioma in the differential diagnosis of temporal masses, even in the absence of classic neurologic symptoms. Management with resection of the accessible component and observation of the residual disease represents a reasonable approach in elderly patients with complex, multicompartmental involvement.

### Limitations

A limitation of this report is the short imaging follow-up interval. Longer-term surveillance imaging could not be obtained at the time of revision because the patient traveled abroad and elected to defer further treatment and follow-up imaging despite counseling regarding the risk of progression.

## Data Availability

The original contributions presented in the study are included in the article/supplementary material, further inquiries can be directed to the corresponding author/s.
